# Exome sequencing reanalysis identifies a novel likely pathogenic *CFAP54* variant and expands the phenotypic and genotypic spectrum of primary ciliary dyskinesia

**DOI:** 10.3389/fmed.2025.1712038

**Published:** 2025-11-28

**Authors:** Yixuan Li, Wangji Zhou, Wanqing Lu, Qiaoling Chen, Yaqi Wang, Xiaogang Li, Kai-Feng Xu, Xue Zhang, Xinlun Tian, Yaping Liu

**Affiliations:** 1 The State Key Laboratory for Complex, Severe, and Rare Diseases, Center for Rare Diseases, Peking Union Medical College Hospital, Chinese Academy of Medical Sciences, Peking Union Medical College, Beijing, China; 2 State Key Laboratory of Complex Severe and Rare Diseases, Department of Pulmonary and Critical Care Medicine, Peking Union Medical College Hospital, Chinese Academy of Medical Sciences, Peking Union Medical College, Beijing, China; 3 State Key Laboratory of Complex Severe and Rare Diseases, Biobank Facility, National Infrastructures for Translational Medicine, Peking Union Medical College Hospital, Chinese Academy of Medical Sciences, Peking Union Medical College, Beijing, China; 4 State Key Laboratory of Medical Molecular Biology, McKusick-Zhang Center for Genetic Medicine, Institute of Basic Medical Sciences, Chinese Academy of Medical Sciences, Peking Union Medical College, Beijing, China

**Keywords:** primary ciliary dyskinesia, exome sequencing reanalysis, *CFAP54*-related phenotype and genotype, minigene assay, splicing variant

## Abstract

**Background:**

Primary ciliary dyskinesia (PCD) is a rare genetic disorder caused by structural or functional abnormalities of motile cilia, characterized by considerable clinical and genetic heterogeneity. Although exome sequencing (ES) can improve the diagnostic rate of PCD, more than 30% of patients with clinically suspected PCD remain undiagnosed by initial ES. The American College of Medical Genetics and Genomics (ACMG) recommends periodic reanalysis of ES data to increase the diagnostic yield.

**Methods:**

We investigated a 35-year-old female patient with bronchiectasis and a strong clinical suspicion of PCD from Peking Union Medical College Hospital. The patient’s ES data, which had initially yielded negative results in August 2024, was reanalyzed in early 2025 employing a “genotype-phenotype-inheritance pattern” strategy. A minigene assay was conducted to validate the pathogenicity of the identified *CFAP54* splicing variant. Variant pathogenicity was classified according to the ACMG/AMP guidelines.

**Results:**

The patient had a history of rhinitis and neonatal pneumonia. Pulmonary function tests revealed moderate obstructive ventilatory dysfunction. ES reanalysis identified a homozygous variant, *CFAP54* (NM_001306084.2):c.6965 + 5G >A, which was initially classified as a variant of uncertain significance. Minigene assays confirmed that this variant induced exon 50 skipping, resulting in a frameshift and a premature termination codon (loss-of-function). This variant was subsequently reclassified as “Likely Pathogenic”.

**Conclusion:**

This study is the first to describe *CFAP54*:c.6965 + 5G >A and confirm its pathogenicity. This finding brings the total number of *CFAP54*-associated PCD patients to eight and the number of distinct *CFAP54* mutations to twelve, thereby enriching the phenotypic and genotypic spectrum of this gene. Furthermore, this effective strategy of “ES reanalysis + minigene verification” resolved the diagnostic dilemma in initially ES-negative PCD cases, providing a replicable molecular diagnostic framework for similar scenarios.

## Introduction

Primary ciliary dyskinesia (PCD) is a rare genetic disorder caused by structural or functional abnormalities of motile cilia, characterized by significant clinical and genetic heterogeneity. It is predominantly inherited in an autosomal recessive manner, although X-linked recessive and autosomal dominant inheritance have also been reported (1, 2). To date, over 50 PCD-causing genes have been identified worldwide. The vast majority of these genes encode proteins associated with the axoneme of motile cilia or sperm flagella, the ciliary membrane, the basal body, or are key factors involved in proper cilium assembly (3). A small number of other causative genes regulate the processes of motile cilium biogenesis, differentiation, and maturation (4).

As a classic multisystem ciliopathy, PCD primarily manifests as chronic respiratory tract diseases (nose, sinuses, middle ear, and lungs). It can also lead to fertility issues, hydrocephalus (5) and situs inversus or congenital heart defects (6). It is reported that the global prevalence of PCD is at least 1/7554 (7). However, the incidence of PCD varies considerably across different regions, ethnicities, and data sources, with reported rates ranging from 1/10,000 to 1/40,000 (8, 9). Notably, the scarcity of reported PCD cases in China, coupled with the strong phenotypic heterogeneity of the disease, significantly increases the risk of underdiagnosis or misdiagnosis (10).

In clinical practice, the application of exome sequencing (ES) has substantially improved the diagnostic rate for PCD, with reported diagnostic yields ranging between 25% and 40% across different studies (11, 12). While diagnostic rates for PCD using ES have been reported to reach approximately 70% (3, 13), over 30% of patients with a clinical suspicion of PCD remain without a definitive molecular diagnosis following initial ES testing.

The continuous discovery of novel PCD-causing genes, the re-interpretation of Variants of Uncertain Significance (VUS), and advancements in bioinformatics technologies have made the reanalysis of initially negative ES data a crucial strategy for identifying potential pathogenic variants. Reanalysis of ES encompasses two primary approaches: phenotype-driven reanalysis and genotype-driven reanalysis (14). The value of phenotype-driven reanalysis resides in its capacity to conduct targeted reassessment of existing variants by incorporating updated phenotypic correlations, particularly when a patient’ s clinical manifestations or family history are revised (15). This process facilitates the identification of previously overlooked variants that may attain clinical relevance subsequent to refinement or expansion of the phenotypic profile.

Genotype-driven reanalysis derives its value from leveraging newly available evidence at the gene and variant levels, including recently established gene-disease relationships, functional study data, updated population frequency information, and enhanced bioinformatic pipelines (16). It is noteworthy that an improved understanding of the genetic etiology of the patient’ s condition enables the identification of newly reported disease-associated genes and variants that have emerged over time, even when there is no updated phenotypic information. In this study, we detected variants in *CFAP54* through reanalysis, a gene newly listed in OMIM in 2025 as associated with PCD. These two reanalysis strategies are complementary in nature and are highly dependent on sustained collaboration between laboratories and clinicians. Currently, ES data reanalysis is formally recommended by the American College of Medical Genetics and Genomics (ACMG) and can be performed periodically by laboratories based on clinician requests, thereby leveraging the core role of ES technology in the clinical diagnosis of PCD (17).

In recent years, novel PCD-causing genes have been continuously identified, including *CFAP54* (OMIM: 621121). In 2023, Tian et al. reported that biallelic deleterious mutations in *CFAP54* cause severe macrozoospermia and multiple morphological abnormalities of the sperm flagella (MMAF) and non-obstructive azoospermia (NOA) in humans (18). They postulated that *CFAP54* might directly or indirectly regulate the localization/expression of *IFT20*, *IFT52*, *IFT122*, and *SPEF2*, thereby affecting sperm flagella assembly and spermatogenesis. In September of the same year, our group reported two unrelated PCD pedigrees (19), further elucidating the disease association of *CFAP54*. The patients in both families carried compound heterozygous variants in *CFAP54*, whose pathogenicity was confirmed through relevant functional experiments. By generating and phenotypically validating a knock-in mouse model, *CFAP54* was established as a novel PCD-causing gene. Its variants lead to PCD and reproductive abnormalities by reducing mRNA expression and disrupting the structure and function of cilia and sperm flagella, thus expanding the spectrum of PCD pathogenesis. In December 2024, Kai Wohlgemuth et al. identified biallelic variants in *CFAP54* in four unrelated families affected by PCD (20), including nonsense, splice-site, and frameshift mutations, all meeting ACMG criteria for pathogenicity. They discovered that CFAP54 forms a C1d subcomplex of the ciliary central apparatus (CA) with CFAP46 and CFAP74. Variants in *CFAP54* led to the absence of CFAP46 localization in the ciliary axoneme, while variants in *CFAP74* reduced its axonemal localization, collectively disrupting the integrity of the C1d projection.

**FIGURE 1:**
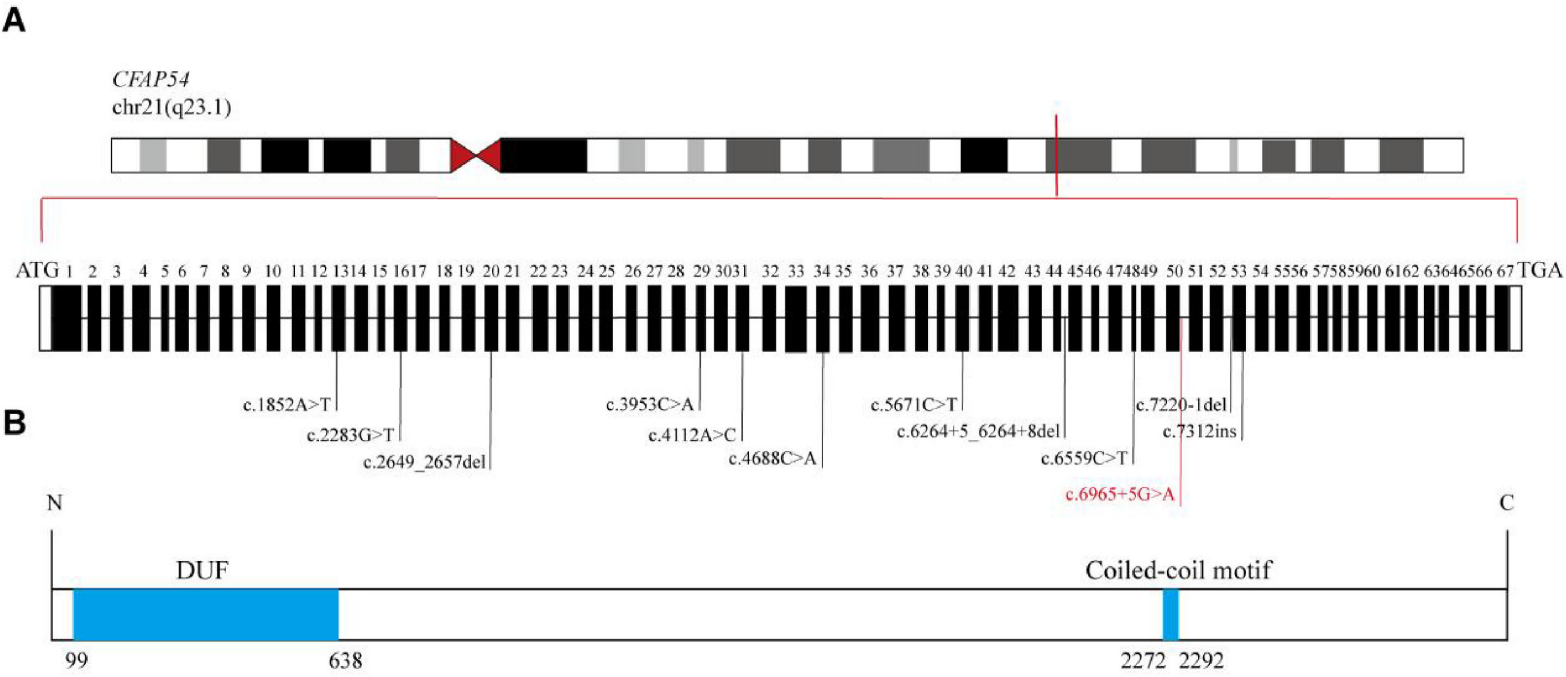
Mutation spectrum and schematic diagram of the functional domains of *CFAP54.* **(A)** Summary of the *CFAP54* mutation spectrum reported prior to this study. The variant identified in this study is highlighted in red. **(B)** Schematic representation of the functional domains of the CFAP54 protein, which includes a Coiled-coil motif and a domain of unknown function (DUF).

As of the initiation of this study, a total of 7 cases of *CFAP54*-related PCD have been reported globally, cumulatively involving 11 pathogenic variants (Figure 1A). This study reports a case with a rare *CFAP54* variant identified through ES data reanalysis, with its pathogenicity confirmed by functional validation, thereby supplementing the mutational spectrum and evidence for the pathogenic mechanisms associated with this gene.

## Materials and methods

### Patients and clinical materials

**FIGURE 2:**
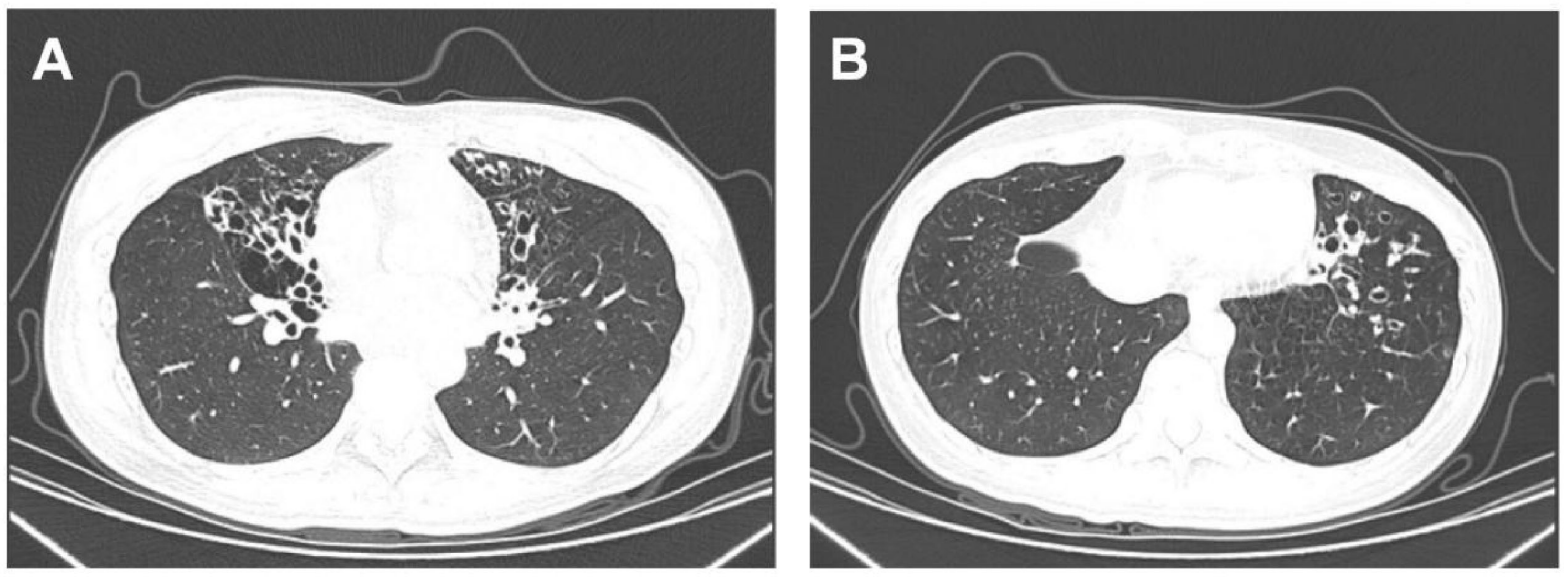
Chest CT of the proband. **(A)** Chest CT showed multiple cystic and cylindrical bronchiectasis with wall thickening in the right middle lobe and left lingular lobe. **(B)** Chest CT showed multiple cystic and cylindrical bronchiectasis with wall thickening in the left lower lobe.

A 35-year-old female presented to the outpatient clinic of the Department of Pulmonary and Critical Care Medicine, Peking Union Medical College Hospital, due to bronchiectasis. At the age of 17, she underwent a chest computed tomography (CT) scan for cough and expectoration, which revealed bronchiectasis. Since then, her symptoms have been stable, and she denied a history of recurrent respiratory infections with normal exercise tolerance. A recent chest CT scan, performed due to aggravated cough and expectoration, revealed multiple bronchiectasis in both lungs (Figure 2). She was a full-term infant with a history of neonatal pneumonia and has had rhinitis for more than 10 years. She conceived naturally and has one son. Her parents are non-consanguineous and have no respiratory symptoms. Physical examination revealed moist rales in the left lower lung, with no situs inversus. Her nasal exhaled nitric oxide level was 149.6 nl/min, which was within the normal range. Pulmonary function tests revealed moderate obstructive ventilatory dysfunction, with a forced expiratory volume in 1 s (FEV_1_) of 1.9 L, FEV_1_ percentage of predicted value of 58%, and FEV_1_/forced vital capacity ratio of 56.83%. Sputum culture yielded *Pseudomonas aeruginosa*. Her antinuclear antibody, rheumatoid factor, immunoglobulin, lymphocyte count, eosinophil count, *Aspergillus*-specific IgE, and sweat chloride test were all normal, essentially ruling out bronchiectasis caused by connective tissue diseases, immunodeficiency, allergic bronchopulmonary aspergillosis, and cystic fibrosis.

Peripheral blood samples were collected from the proband for subsequent experimental analysis. All study procedures were approved by the Institutional Review Board of Peking Union Medical College Hospital (I-23PJ390), and written informed consent was obtained from the patient.

### Sample processing and ES sequencing

Genomic DNA was extracted from the patient’s EDTA-anticoagulated venous blood sample using the QIAamp DNA Blood Mini Kit (QIAGEN, Hilden, Germany) according to the manufacturer’s standard protocol. Exome capture was performed on the gDNA using the Agilent SureSelect Human All Exon V6 probe (Agilent, USA), followed by ES.

### Multi-dimensional reanalysis strategy for ES data

Genotype-driven analysis: We selected variants classified as pathogenic or likely pathogenic within the Phoenix platform^1^. Additionally, potentially pathogenic variants, including rare loss-of-function (LOF) variants and ClinVar/HGMD pathogenic variants, with a MAF <0.01 and a variant allele fraction (VAF) >0.2, were included. Following manual curation (e.g., assessing phenotype-inheritance mode compatibility), the VCF file was imported into the Franklin platform^2^ for secondary analysis. Results from both platforms were compared and integrated.

Inheritance mode-driven analysis: Filtering criteria were set based on inheritance patterns, including autosomal recessive, autosomal dominant, X-linked recessive, and X-linked dominant. Variants were filtered in conjunction with OMIM disease phenotypes and Human Phenotype Ontology terms. The match between the variant’s implied inheritance pattern and the patient’s clinical phenotype was assessed manually.

Phenotype-driven analysis: The Human Phenotype Ontology includes the following terms: HP:0002110 Bronchiectasis; HP:0012384 Rhinitis; HP:0012735 Cough; and HP:0031245 Productive Cough. Based on the Phoenix clinical phenotype results, variants with a phenotype score of H (or a custom threshold) and MAF <0.01 were selected, and their pathogenicity was preliminarily assessed according to the ACMG/AMP guidelines. Copy number variations (CNVs) with an AnnotSV (ClinGen) score >0.5 were also retained, with a focus on candidate genes associated with PCD and respiratory diseases.

### Candidate variant filtering and sanger validation

Candidate variants were filtered against public databases (1000 Genomes, ExAC, gnomAD, and ESP) (21–23) to exclude those with a minor allele frequency (MAF) ≥0.01. Specific primers were designed using Primer 5 software, and Sanger sequencing was employed to validate the shortlisted candidate variants, confirming their presence.

### Minigene plasmid construction

To validate the pathogenicity and splicing impact of the *CFAP54* (NM_001306084.2):c.6965 + 5G > A variant, a minigene assay was performed using the pCAS2 vector (with a multiple cloning site located between the vector’s inherent exon A and B). Given the variant’s location in intron 50, genomic DNA fragments from both a control individual and the patient were PCR-amplified. These fragments spanned a complete region including exon 49, intron 49, exon 50, and partial segments of intron 48 (542 bp) and intron 50 (718 bp), resulting in a total fragment length of 3383 bp. The amplified fragments were directionally cloned into the *Bam*HI-digested and linearized pCAS2 vector using the In-Fusion HD Cloning Kit (Takara, Japan). This process generated wild-type (c.6965 + 5G) and mutant (c.6965 + 5A) plasmids. The primer sequences used were:

*CFAP54*-*Bam*HI-F: AAGAAGTGCAGGATCTTTGTTGTA AGAATCAGCTTCAGCC
*CFAP54*-*Bam*HI-R: CTTTCTCCTGGGATCAACAGTTCAG TGCAATATGATGC

### Transfection of minigene plasmids and cDNA sequencing

HEK293 cells were cultured in 6-well plates (1 × 10^6^ cells per well) and transfected with the recombinant plasmids using Lipofectamine 3000 (Life Technologies, CA, USA). Transfections were performed in triplicate for each plasmid (wild-type, mutant, and empty vector), with the empty vector serving as a negative control to exclude interference from the vector’s own sequences on splicing outcomes. Forty-eight hours post-transfection, cells were harvested, and total RNA was extracted using TRIzol reagent (Gibco, San Francisco, CA, USA). RNA purity and concentration were measured using a NanoDrop 2000 spectrophotometer (Thermo, Lithuania) to ensure quality for subsequent steps. Qualified RNA was reverse-transcribed into cDNA using the PrimeScript RT Master Mix (Takara, Japan). Reverse transcription-polymerase chain reaction (RT-PCR) primers were designed to span the vector exons and the target gene exons, with the forward primer situated between vector exon A and target exon 49, and the reverse primer within vector exon B, to amplify the splicing products. The primer sequences were:

*CFAP54*-pCAS2-RT-F: GCTGCTGCTGCTGGCTGGGTCGA TG
*CFAP54*-pCAS2-RT-R: AGCCCCGAGCAGGACGTGGGTA AGG

The RT-PCR products were analyzed by agarose gel electrophoresis and UV imaging to assess differences in fragment size. Sanger sequencing was subsequently used to precisely characterize the type of splicing abnormality.

### Pathogenicity analysis

All variants were classified according to the ACMG/AMP guidelines (24). These criteria are categorized into two groups: Pathogenic or Likely Pathogenic (P/LP) and Benign or Likely Benign (B/LB). Variants are classified as “VUS” when criteria are conflicting or not met. The standardized evaluation encompasses various data types, including population frequency, computational predictions, functional evidence, familial cosegregation data, *de novo* variant data, allelic data, and clinical relevance.

## Results

### ES data reanalysis

The patient developed bronchiectasis during childhood and had a history of rhinitis and neonatal pneumonia. According to the PCD diagnostic guidelines of the European Respiratory Society (25), these clinical manifestations strongly suggest a diagnosis of PCD.

**FIGURE 3:**
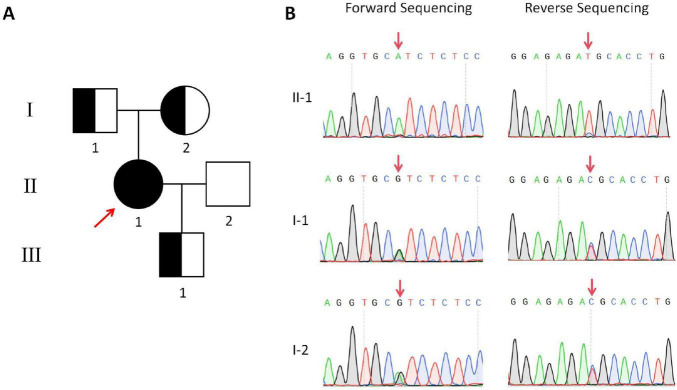
Pedigree of the proband and Sanger sequencing chromatograms of the variant. **(A)** The proband is indicated by a red arrow. Her parents, husband, and son show no respiratory-related phenotypes. **(B)** Bidirectional Sanger sequencing spanning the variant site (*CFAP54*:c.6965 + 5G > A) in the proband (II-1) confirmed a homozygous variant. Both of her parents (I-1, I-2) were identified as heterozygous carriers of this variant. While her son (III-1) did not undergo Sanger validation, he is necessarily a carrier.

The patient underwent singleton ES in August 2024 and the initial report did not identify any variants that could explain the patient’s phenotype. In early 2025, the laboratory initiated a reanalysis of the patient’s ES data. A novel PCD-associated homozygous variant, *CFAP54* (NM_001306084.2):c.6965 + 5G > A, was identified via a genotype-driven reanalysis strategy (*CFAP54* was included in OMIM as a pathogenic gene for PCD in March 2025). This homozygous variant was subsequently confirmed by Sanger sequencing, and it was inherited from both parents, who are both heterozygous carriers (Figure 3). The variant is located at a non-canonical splice site. It was absent from the 1000 Genomes Project and the Exome Aggregation Consortium (ExAC) databases, and its allele frequency in the East Asian population in gnomAD was 0.000, fulfilling the PM2 criterion (Supporting evidence of pathogenicity). Computational predictions yielded a SpliceAI score of 0.19 and a moderate phyloP conservation score of 0.82, suggesting a potential impact on splicing and moderate evolutionary conservation of the affected region. According to the ACMG guidelines, the variant was initially classified as VUS. This variant is located near exon 50 of *CFAP54*, which encodes part of a coiled-coil motif (Figure 1B). Therefore, the c.6965 + 5G > A variant is predicted to have the potential to disrupt the normal conformation of this coiled-coil domain, and thus potentially affect the overall function of the protein.

### Minigene functional validation

**FIGURE 4:**
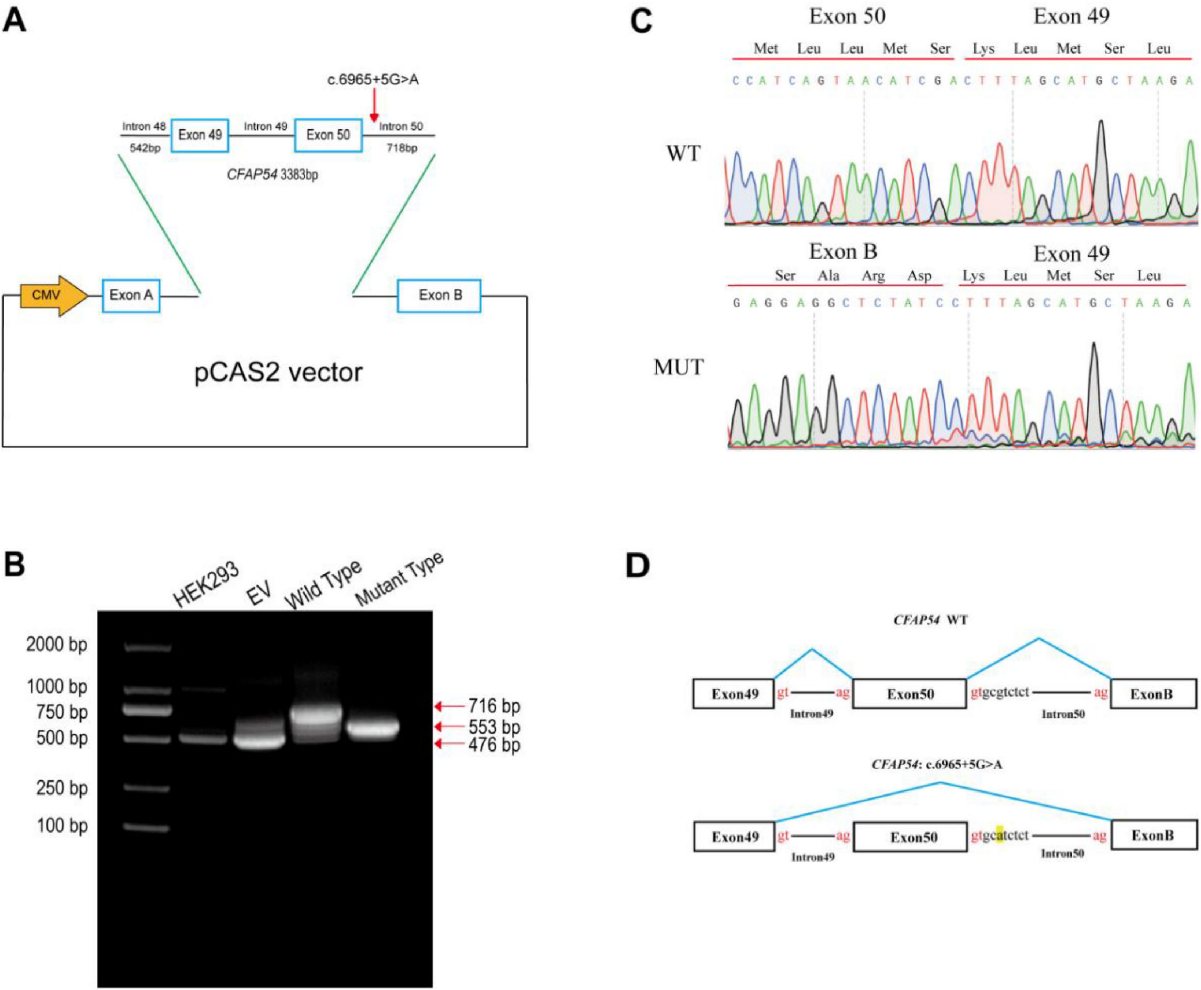
Construction of the Minigene vector and experimental results. **(A)** Schematic diagram of the mutant Minigene vector construction. The plasmid backbone is pCAS2, with *Bam*HI used as the restriction site. The inserted fragment was amplified by PCR from the proband’s peripheral blood DNA. This fragment spans a complete genomic region including exon 49, intron 49, exon 50, plus partial segments of intron 48 (542 bp) and intron 50 (718 bp), with a total length of 3383 bp. The mutation site is indicated by a red arrow. **(B)** Agarose gel electrophoresis results. “HEK293” represents a transfection reagent-only control group, ruling out any effect of the reagent on cellular splicing. “EV” denotes the empty vector control group, excluding any impact of the vector itself on splicing. “Wild Type” represents the group transfected with the vector containing the PCR fragment from a control individual. “Mutant Type” represents the group transfected with the vector containing the PCR fragment from the patient. The amplified transcript from the wild-type plasmid is approximately 716 bp, while the mutant plasmid produces a shorter transcript of approximately 553 bp. **(C)** Reverse-strand Sanger sequencing chromatograms of the transfection products. “WT” represents the group with the control insert; “MUT” represents the group with the patient insert. **(D)** Schematic diagram illustrating the splicing patterns.

As the *CFAP54*:c.6965 + 5G > A variant is predicted to affect splicing, an *in vitro* minigene assay was performed to investigate its impact on RNA splicing. The constructed wild-type (c.6965 + 5G) and mutant (c.6965 + 5A) minigene plasmids were transfected into HEK293 cells (Figure 4A). Total RNA was extracted 48 h post-transfection and analyzed by RT-PCR. Agarose gel electrophoresis revealed a transcript of approximately 716 bp for the wild-type plasmid. In contrast, the mutant plasmid produced a significantly shorter transcript of approximately 553 bp, indicating an aberrant splicing pattern (Figure 4B). Sanger sequencing confirmed that the mutant transcript resulted from the complete skipping of exon 50 (161 bp in length) (Figures 4C, D). This 161-nucleotide deletion resulted in an out-of-frame shift in the reading frame, introducing a premature termination codon (PTC) downstream. This molecular mechanism is consistent with a LOF effect.

### Pathogenicity assessment

Integrating the evidence from the functional minigene assay, the pathogenicity of the *CFAP54* (NM_001306084.2):c.6965 + 5G > A variant was re-evaluated. According to the ACMG guidelines, its initial classification was VUS, supported by the PM2 criterion. The minigene experiment provided definitive functional evidence that the variant causes exon 50 skipping and introduces a PTC, leading to a loss of function of CFAP54 protein. This finding fulfills the PVS1 (Null variant in a gene where LOF is a known mechanism of disease; and non-canonical splice variants where RNA analysis confirms aberrant transcription.) criterion of the ACMG/AMP guidelines (26). With the addition of the PVS1 evidence, the variant’s pathogenicity classification was upgraded to Likely Pathogenic (Table 1).

**TABLE 1:** Variant assessment information for the proband.

Information	*CFAP54* variant
cDNA alteration	NM_001306084.2:c.6965 + 5G > A
Zygosity	Homozygous
Variant type	Splicing variant
1000 Genomes Project	N/A
Exome Aggregation Consortium	N/A
East Asians in gnomAD	0.000
Splice AI	Donor Loss 0.19; Donor Gain 0.19
phyloP	Conserved (0.82)
Initial ACMG criteria	PM2
Initial pathogenicity	VUS
Added ACMG criteria	PVS1
Pathogenicity	Likely pathogenic

VUS, variant of uncertain significance.

## Discussion

In this study, reanalysis of ES data from a previously negative PCD case led to the identification of a homozygous non-canonical splice variant in *CFAP54* (NM_001306084.2:c.6965 + 5G > A). This variant was not reported initially, primarily because the patient’s sample was first submitted for testing in August 2024, before *CFAP54* was officially listed as a PCD-causing gene in the OMIM database^3^. This finding directly underscores the critical value of dynamically updated disease knowledge bases and periodic ES reanalysis in overcoming the inherent lag in PCD diagnosis.

Since CFAP54 is a structural ciliary protein with extremely low expression in human peripheral blood, validating the splicing effect directly from the patient’s blood sample was not feasible. Consequently, we employed a minigene assay to simulate the splicing process *in vitro*. Our experiment confirmed that the c.6965 + 5G > A variant causes exon 50 skipping in *CFAP54*, leading to a frameshift and the introduction of a PTC, ultimately resulting in a loss of protein function. Our functional validation not only fills the evidence gap for the *CFAP54*:c.6965 + 5G > A variant but also brings the total number of reported *CFAP54*-associated PCD patients to 8 and the number of distinct mutations to 12 (18–20), thereby enriching the genotypic and phenotypic spectrum of this gene Furthermore, by identifying a pathogenic variant through the reanalysis of initially negative ES data, this study highlights the pivotal role of reanalyzing ES data in combination with functional experiments to resolve diagnostic dilemmas in clinically suspected cases. It provides a generalizable technical approach for the molecular diagnosis of genetically heterogeneous disorders like PCD.

*CFAP54*, a recently identified PCD-causing gene, encodes a protein that is integral to the formation of the C1d subcomplex within the ciliary axoneme. It works in conjunction with CFAP46 and CFAP74 to maintain ciliary structural integrity (20). The c.6965 + 5G > A variant identified is this study is located at a non-canonical splice site flanking exon 50 and leads to a loss of function through aberrant splicing. This pathogenic mechanism is consistent with those of previously reported *CFAP54* variants (e.g., nonsense, frameshift), all of which disrupt the structure and function of the ciliary axoneme, thereby leading to PCD. Notably, *CFAP54* was initially associated with male infertility due to MMAF and NOA (18). However, the patient in our study is a female and conceived naturally, suggesting potential sex-dependent differences or tissue specificity in the phenotypic expression of *CFAP54* variants (27), which aligns with the multi-system nature of PCD-related genes. Additionally, the patient presented with bronchiectasis, rhinitis, and a history of neonatal pneumonia, but no situs inversus, and her nasal exhaled nitric oxide level was 149.6 nl/min, which fell within the normal range. This phenotype aligns with those of previously reported cases (18–20) (Table 2). Notably, embryonic nodal cilia, which have a 9 + 0 structure (characterized by nine peripheral microtubule doublets and absent central microtubules) (28) regulate organ positioning and lack the CA. This explains why these patients display situs solitus rather than situs inversus. Therefore, variants in genes such as *CFAP46*, *CFAP54*, *CFAP74*, and *CFAP221* cause C1d defects of the central microtubules (20). Affected patients exhibit normal organ situs, as well as normal nNO, transmission electron microscopy (TEM), and high-speed video microscopy analysis (HSVA) results (Table 2). For such individuals, genetic testing is particularly crucial and represents almost the only diagnostic method available.

**TABLE 2:** Summary of clinical manifestation and Supplementary Examination for all current *CFAP54*-related PCD patients.

	Clinical manifestation					Supplementary examination			
Patients	Cough, sputum production and bronchiectasis	Otitis media	Situs inversus	Fertility impairment	Neonatal symptoms	nNO (nl/min)	TEM	HSVA	Genetic analysis
1 (19)	+	/	-	/	/	80	Respiratory cilia: normal sperm flagellum: the “9 + 2” microtubule structure is incomplete	/	c.2649_2657delinC (p.E883Dfs*47); c.7312_7313insCGCAGGCTGAATTCTTGG (p.T2438delinsTQAEFLA)
2 (19)	+	/	/	/	Neonatal pneumonia	/	/	/	c.2649_2657delinC (p.E883Dfs*47); c.7312_7313insCGCAGGCTGA ATTCTTGG (p.T2438delinsTQAEFLA)
3 (19)	+	/	-	-	/	114	/	/	c.4112A > C (p.E1371A); c.6559C > T (p.P2187S)
4 (20)	+	/	-	/	/	/	/	/	c.4688C > A (p.Ser1563Ter), homozygous
5 (20)	+	/	-	/	/	182.3	Normal	Normal	c.3953C > A (p.Ser1318Ter); c.6264 + 5_6264 + 8del (p.Gly2028_Ile2088del)
6 (20)	+	/	-	/	Neonatal respiratory distress with pneumonia	176.4	Normal	Normal	c.2283G > T (p.Lys761Asn_Arg762insTer7); c.5671C > T (p.Arg1891Ter)
7 (20)	+	/	-	/	Neonatal respiratory distress	/	/	Normal	c.1852A > T (p.Lys618Ter); c.7220-1delG
8^#^	+	-	-	-	Neonatal pneumonia	149.6	/	/	c.6965 + 5G > A, homozygous

nNo, nasal nitric oxide; TEM, transmission electron microscopy; HSVA, high-speed video microscopy analysis; “ + “ means positive; “-” means negative; “/” means unknown; “#” means the patient in this study.

This study has three main limitations that should be objectively acknowledged: (1) The absence of TEM of cilia or HSVA to directly validate ciliary ultrastructural and functional defects. However, normal results would be expected, as previously reported in *CFAP54*-related PCD. (2) Although the minigene assay effectively models splicing effects, this *in vitro* system cannot fully replicate the complex *in vivo* splicing environment. Factors such as cell- or tissue-specific splicing factors, transcription kinetics, chromatin state, isoform balance, and intron size are not perfectly captured (29). Nevertheless, it is important to emphasize that minigene assays remain a robust and widely accepted surrogate method for validating the pathogenicity of splicing variants when patient-derived affected tissues (e.g., respiratory epithelial cells) are unavailable, and their results hold significant clinical value, as recognized in the field (30).

## Conclusion

Through ES reanalysis and functional validation, this study first identified the likely pathogenic *CFAP54* variant (c.6965 + 5G > A) in a PCD patient. This finding brings the total number of reported CFAP54-related PCD cases to 8 and distinct mutations to 12, thereby broadening the genotypic and phenotypic spectrum of CFAP54-associated PCD. This case also reaffirms that for PCD patients with an initial negative genetic test result, ES data reanalysis guided by clinical phenotype, supplemented by targeted functional experiments, constitutes an effective strategy for improving diagnostic yield. With the continuous discovery of novel disease-causing genes and advancements in functional validation technologies, the molecular diagnostic rate for PCD is expected to increase further, laying a solid foundation for precision medicine in the near future.

## Data Availability

The raw data supporting the conclusions of this article will be made available by the authors, without undue reservation.

## Footnotes

1
https://phoenix.imperialgene.com

2
https://franklin.genoox.com

3
https://www.omim.org
